# Quantitative analysis of lung elastic fibers in idiopathic pleuroparenchymal fibroelastosis (IPPFE): comparison of clinical, radiological, and pathological findings with those of idiopathic pulmonary fibrosis (IPF)

**DOI:** 10.1186/1471-2466-14-91

**Published:** 2014-05-28

**Authors:** Noriyuki Enomoto, Hideki Kusagaya, Yoshiyuki Oyama, Masato Kono, Yusuke Kaida, Shigeki Kuroishi, Dai Hashimoto, Tomoyuki Fujisawa, Koshi Yokomura, Naoki Inui, Yutaro Nakamura, Takafumi Suda

**Affiliations:** 1 Second Division, Department of Internal Medicine, Hamamatsu University School of Medicine, 1-20-1 Handayama, Hamamatsu 431-3192, Japan; 2 Department of Internal Medicine, Enshu Hospital, 1-1-1 Chuo, Hamamatsu 430-0929, Japan; 3 Department of Respiratory Medicine, Seirei Mikatahara General Hospital, 3453 Mikataharacho, Hamamatsu 433-8558, Japan

**Keywords:** Elastic fiber, Pleuroparenchymal fibroelastosis, Idiopathic pulmonary upper lobe fibrosis, Usual interstitial pneumonia, Idiopathic pulmonary fibrosis, Quantitative analysis

## Abstract

**Background:**

The pathological appearance of idiopathic pleuroparenchymal fibroelastosis (IPPFE) with hematoxylin-eosin staining is similar to that of usual interstitial pneumonia (UIP) in patients with idiopathic pulmonary fibrosis (IPF). The amount of elastic fibers (EF) and detailed differences between IPPFE and IPF have not been fully elucidated. The aim of this study was to quantify the EF and identify the differences between IPPFE and IPF.

**Methods:**

We evaluated six patients with IPPFE and 28 patients with IPF who underwent surgical lung biopsy or autopsy. The patients’ clinical history, physical findings, chest high-resolution computed tomography (HRCT) findings, and pathological features of lung specimens were retrospectively evaluated. The amounts of EF in lung specimens were quantified with Weigert’s staining using a camera with a charge-coupled device and analytic software in both groups.

**Results:**

Fewer patients with IPPFE than IPF had fine crackles (50.0% vs. 96.4%, p = 0.012). Patients with IPPFE had a lower forced vital capacity (62.7 ± 10.9% vs. 88.6 ± 21.9% predicted, p = 0.009), higher consolidation scores on HRCT (1.7 ± 0.8 vs. 0.3 ± 0.5, p < 0.0001), lower body mass indices (17.9 ± 0.9 vs. 24.3 ± 2.8, p < 0.0001), and more pneumothoraces than did patients with IPF (66.7 vs. 3.6%, p = 0.002). Lung specimens from patients with IPPFE had more than twice the amount of EF than did those from patients with IPF (28.5 ± 3.3% vs. 12.1 ± 4.4%, p < 0.0001). The amount of EF in the lower lobes was significantly lower than that in the upper lobes, even in the same patient with IPPFE (23.6 ± 2.4% vs. 32.4 ± 5.5%, p = 0.048). However, the amount of EF in the lower lobes of patients with IPPFE was still higher than that of patients with IPF (23.6 ± 2.4% vs. 12.2 ± 4.4%, p < 0.0001).

**Conclusion:**

More than twice the amount of EF was found in patients with IPPFE than in those with IPF. Even in the lower lobes, the amount of EF was higher in patients with IPPFE than in those with IPF, although the distribution of lung EF was heterogeneous in IPPFE specimens.

## Background

Idiopathic pleuroparenchymal fibroelastosis (IPPFE) is a rare disease that was recently classified as a rare idiopathic interstitial pneumonias (IIPs) together with idiopathic lymphoid interstitial pneumonia in an official American Thoracic Society (ATS)/European Respiratory Society (ERS) statement on the international multidisciplinary classification of the IIPs [1]. IPPFE was first described by Frankel et al. in 2004 [2], which showed upper lobe-predominant volume loss, pleural thickening, and prominent subpleural fibroelastosis [2]. IPPFE has clinical, radiological, and pathological features similar to those of idiopathic pulmonary upper lobe fibrosis (IPUF), which was first reported in a Japanese paper by Amitani et al. in 1992 [3]. These two disorders are considered to be within the same spectrum [4].

The pathological features of IPPFE, which include dense subpleural fibroelastosis on elastic staining, are quite specific for this disorder. However, these pathological features on hematoxylin-eosin (HE) staining, including perilobular collagen deposition with abrupt transition to underlying normal parenchyma and fibroblastic foci, are similar to those of usual interstitial pneumonia (UIP) in patients with idiopathic pulmonary fibrosis (IPF) [5]. The UIP-pattern has also been found in the lower lobes of patients with IPPFE [6], and patients with IPPFE are sometimes misdiagnosed with IPF because of their similar pathological findings on HE staining [7]. However, the precise differences between IPPFE/IPUF and IPF have not yet been studied.

All organs contain fibrous connective tissue, which comprises collagen, reticular, and elastic fibers (EF). The proportion of collagen and elastic fibers determines an organ’s physical flexibility and elasticity. In patients with lung fibrosis, the increased proportion of EF within fibrotic tissue reduces compliance and makes the lungs stiff. We recently reported that an increased amount of EF in surgical lung biopsy specimens is an independent predictor of a poor prognosis in patients with IPF [8]. Accumulation of dense EF in the subpleural parenchyma is another specific pathological feature of IPPFE [2]. However, the details regarding the amount and distribution of EF in patients with IPPFE remain unknown, and the difference in the amount of EF between IPPFE and IPF has not been quantitatively evaluated.

In the present study, we compared the clinical, radiological, and pathological findings of IPPFE with those of IPF. In addition, we quantified the amount of EF in lung specimens from patients with IPPFE or IPF using a camera with charge-coupled device (CCD) and analytic software. IPPFE lung specimens had more than twice the amount of EF found in IPF lung specimens. Furthermore, whereas the distribution of lung EF was quite heterogeneous in IPPFE specimens, the amount of EF in the lower lobes was still higher in patients with IPPFE than in those with IPF.

## Methods

### Study population

**Figure 1:**
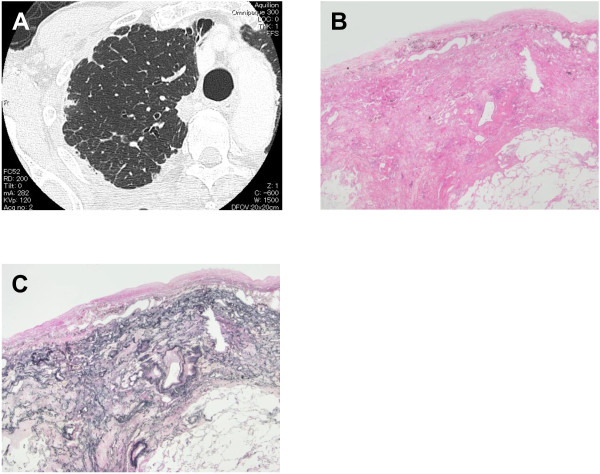
**Representative findings of high-resolution computed tomography (HRCT) and surgical lung biopsy specimens of a patient with idiopathic pleuroparenchymal fibroelastosis (IPPFE).** HRCT shows pleural thickening and subpleural consolidation opacities in the upper lobes **(A)**. A lung section stained with hematoxylin and eosin shows subpleural fibrosis with an abrupt transition to normal lung parenchyma and fibroblastic foci, similar to that seen in usual interstitial pneumonia (**B**, ×12.5). Pleural fibrosis was also seen. A lung specimen with Elastica van Gieson staining demonstrates deposition of dense elastic fibers (elastosis) in a subpleural fibrotic lung lesion (**C**, ×12.5).

Six consecutive patients with IPPFE who underwent surgical lung biopsy or autopsy in our hospital or affiliated hospitals from 2010 to 2011 were evaluated. The pathological diagnosis of IPPFE was based on a previous report by Frankel et al. [2]. Briefly, the diagnosis of IPPFE was based on the presence of prominent subpleural fibroelastosis, sparing of the parenchyma distant from the pleura, and a relatively abrupt border between the fibroelastosis and the underlying normal parenchyma (Figure 1B and C). Clinical, radiological, and pathological data of these patients were retrospectively reviewed. Patients who met the criteria for any connective tissue disorders were excluded from this study.

Twenty-eight consecutive patients with IPF who underwent surgical lung biopsy in our hospital from 1997 to 2007 were also evaluated in this study. The initial diagnosis of IPF was based on the latest diagnostic criteria of that period. All 28 patients also met the 2011 IPF consensus criteria of the ATS, ERS, Japanese Respiratory Society (JRS), and Latin American Thoracic Association (ALAT) [5]. The histologic features of UIP were based on a previously published reports [5,9]. No lung specimens had a “not UIP pattern” as defined by the IPF consensus criteria (i.e., presence of hyaline membranes, organizing pneumonia, granulomas, marked interstitial inflammatory cell infiltrate away from honeycombing, or predominant airway-centered changes) [5]. The study protocol was approved by the Ethics Committee of Hamamatsu University School of Medicine (approval number 24-167).

### Quantification of EF

**Figure 2:**
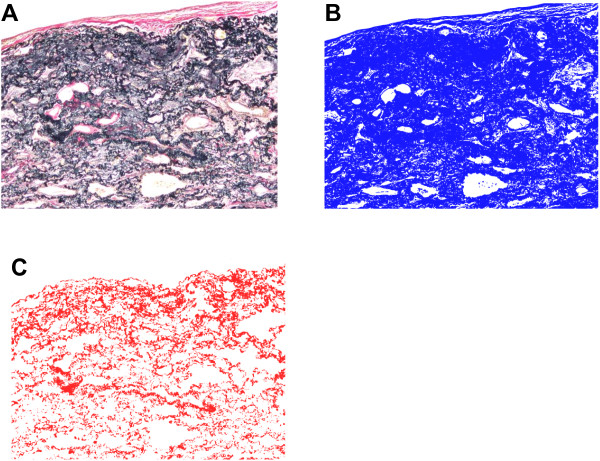
**Images of lung sections in a patient with idiopathic pleuroparenchymal fibroelastosis (IPPFE).** The images were made using a microscope with a camera equipped with a charge-coupled device and image analysis software. Surgical lung specimens were stained with Elastica van Gieson **(A)**, and imaged lesions at 40x magnification in all specimens were captured. Images were changed to gray scale, then binarized to detect the area containing the fibrotic lung lesion using software (**B**, blue area). The images were binarized with another threshold to estimate elastic fibers (**C**, red area). The concentration of elastic fibers (EF score) in the fibrotic area was calculated by dividing the pixel number of elastic fibers (red area) by that of the target fibrotic lung lesion (blue area). Surgical biopsy images from one patient with an EF score of 27.5% are shown.

All available lung specimens were examined. Samples were fixed in neutral buffered 10% formalin and embedded in paraffin. Staining was performed on 4-μm sections mounted on glass slides. Sections were stained by HE or Weigert’s resorcin-fuchsin solution. Images of sections stained with Weigert’s elastic staining were collected using a microscope with a CCD camera (DP21; Olympus, Tokyo, Japan). Images of fibrotic lesions at × 40 magnification were obtained from all slides (Figure 2A). For each image, the area occupied by EF was quantified using imaging software (Image J; National Institutes of Health, Bethesda, MD and Photoshop Elements; Adobe, San Jose, CA), according to our previous report [8]. Images were converted to gray scale, then binarized with two different thresholds to quantify the area of the whole fibrotic lung lesion (Figure 2B, blue area) and the area of EF (Figure 2C, red area). The proportion of EF in the fibrotic interstitial area was calculated by dividing the number of pixels containing EF (red area) by that of the whole fibrotic lesion (blue area). For each patient, the mean proportion of EF in all histological images was calculated and expressed as a percentage (the EF score).

### Evaluation of other pathological findings

The investigators examining the histological specimens also evaluated the collagen deposition, cellularity, and organization in the air spaces. The severity of each of these findings was scored on a scale from 0 to 3 (0, none; 1, mild; 2, moderate; 3, severe). The presence of honeycombing, lymphoid hyperplasia, and pleuritis was also recorded. These findings were reviewed by two observers and the agreement rates between the observers were evaluated by weighted-kappa coefficients. The coefficients ranged from 0.34 to 0.68. When the score differed between the observers, a consensus was reached after discussion.

### Evaluation of high-resolution computed tomography findings

The extent of lung fibrosis was measured using high-resolution computed tomography (HRCT) on slices taken at the tracheal bifurcation, the base of the lower lobes, and at the midpoint. The extent of fibrosis in each lobe was scored using the following system: 0, none; 1, 1–10%; 2, 11–25%; 3, 26–50%; 4, 51–75%; and 5, 76–100%. The sum of the scores from five lobes (0–25) was used to express the extent of fibrosis throughout each patient’s lungs. The severity of ground-glass opacity and consolidation was scored using the same scale as that used for the pathologic evaluation. Finally, the presence of honeycombing on HRCT was also evaluated. These findings were reviewed by two observers; the agreement rates between them were evaluated by weighted-kappa coefficients. The coefficients ranged from 0.34 to 0.83. When the score differed between the observers, a consensus was reached after discussion.

### Data collection

Clinical data, including sex, age, smoking history, symptoms, treatment, and survival were obtained from the patients’ medical records. Laboratory findings, pulmonary function tests results, and bronchoalveolar lavage (BAL) data at the time of surgical lung biopsy or before autopsy were also recorded.

### Statistical analysis

Statistical analyses were performed using StatView J-4.5 (SAS Institute, Inc., Cary, NC, US). Categorical data were compared using the chi-square test or Fisher’s exact probability test for independence. Continuous data were compared using a paired or unpaired Student’s t-test. Discontinuous data were compared using Mann-Whitney’s U-test. The relationship between the EF score and serial data was analyzed using Pearson’s correlation coefficient, and that between the EF score and discrete variable data was analyzed using Spearman’s rank correlation coefficient. The overall survival of each group was estimated by Kaplan-Meier curves. The log-rank test was used to compare survival between the two groups. All tests were two-sided, and statistical significance was defined as p < 0.05.

## Results

### Clinical characteristics of patients with IPPFE

Six consecutive patients with IPPFE who underwent surgical lung biopsy or autopsy were evaluated. One patient was autopsied after acute exacerbation of lung disease. Antinuclear antibody was positive at 1:40 in three patients, and rheumatoid factor was positive in two patients. Anti-cyclic citrullinated peptide antibody was positive in one patient. Anti-double-strand DNA antibody and anti-cardiolipin β2 glycoprotein 1 antibody were positive in one patient. However, no patients met the criteria for any connective tissue disorders. No patients had a family history of interstitial lung disease or a history of dust exposure. One patient had undergone chemotherapy for gingival carcinoma. Although repeated bacterial pneumonia had occurred in one patient, no patients had a history of fungal infection such as pulmonary aspergillosis. Refractory pneumothorax appeared shortly after the surgical lung biopsy in one patient.

Representative HRCT and surgical lung biopsy findings specimens from one patient with IPPFE are shown in Figure 1. HRCT showed pleural thickening and subpleural consolidation opacities in the upper lobes (Figure 1A). An HE-stained lung section revealed subpleural fibrosis with abrupt transition to underlying normal lung parenchyma and fibroblastic foci, similar to UIP (Figure 1B). Pleural fibrosis was also seen. Deposition of dense EF (elastosis) in subpleural fibrotic lung lesions, which is a specific histological finding of IPPFE, was seen on a section with Elastica-van Gieson (EVG) staining (Figure 1C).

### Comparison between IPPFE and IPF: clinical characteristics

The clinical characteristics of patients with IPPFE and IPF are compared in Table 1. Five of the six patients with IPPFE were male, and their mean age of the six patients was 69.2 years. The observation period was 39.8 ± 29.2 (mean ± SD) months. Patients with IPPFE had a higher proportion of never-smokers (50% vs. 7.1%, p = 0.011) and a lower mean body mass index (17.9 vs. 24.3 (mean), p < 0.0001) than did patients with IPF. Fewer patients with IPPFE than IPF had fine crackles at the time of diagnosis (50.0% vs. 96.4%, p = 0.012). In addition, patients with IPPFE had more pneumothoraces during the observation period (66.7% vs. 3.6%, p = 0.002). Refractory pneumothorax appeared shortly after surgical lung biopsy in one patient with IPPFE. Although the proportion of patients who underwent medical check-ups to detect interstitial pneumonia was not different between the two groups, the period from detection of interstitial pneumonia to acquisition of lung specimens was significantly longer in patients with IPPFE than in those with IPF (73.3 vs. 29.7 months, p = 0.012). Four of the six patients with IPPFE received therapeutic interventions. Steroid treatment was started in one patient, whilst pirfenidone was administrated to the other three patients. However, these treatments showed no therapeutic effects, and three of the six patients with IPPFE died during the study period. One died of acute exacerbation of IPPFE, and the other two died of chronic disease progression.

**Table 1:** Comparison between idiopathic pleuroparenchymal fibroelastosis (IPPFE) and idiopathic pulmonary fibrosis (IPF): clinical characteristics

	**IPPFE (n = 6)**	**IPF (n = 28)**	**p value**
Sex, male/female	5/1	26/2	0.452*
Age at biopsy, yr	69.2 ± 3.9	61.4 ± 9.9	0.072
Smoking history, current/ex/never	0/3/3	13/13/2	0.011*
Pack-year of smoking	29.3 ± 33.1	66.8 ± 97.4	0.363
Detection by medical check-up, n (%)	3 (50)	21 (75.0)	0.328*
Respiratory symptoms at biopsy, n (%)	6 (100)	19 (67.9)	0.162*
Body mass index, kg/m^2^	17.9 ± 0.9	24.3 ± 2.8	<0.0001
Clubbing of finger, n (%)	1 (16.7)	6 (21.4)	0.999*
Fine crackles, n (%)	3 (50)	27 (96.4)	0.012*
Pneumothorax, n (%)	4 (66.7)	1 (3.6)	0.002*
Acute exacerbation, n (%)	1 (16.7)	6 (21.4)	0.999*
Therapeutic intervention, n (%)	4 (66.7)	15 (53.6)	0.672*
Period from detection of IP until acquisition of lung specimens	73.3 ± 46.1	29.7 ± 34.3	0.012
Observation period, mo	39.8 ± 29.2	67.8 ± 46.7	0.206
Death, n (%)	3 (50.0)	12 (42.9)	0.999*

Data are presented as n, mean ± SD or n (%).

Data were analyzed with Student’s t-test or *Fisher’s exact probability test.

*Abbreviations*: *IPPFE* idiopathic pleuroparenchymal fibroelastosis, *IPF* idiopathic pulmonary fibrosis, *IP* interstitial pneumonia.

### Comparison between IPPFE and IPF: laboratory, physiologic, and BAL data

The laboratory, physiologic, and BAL data between the patients with IPPFE and IPF are compared in Table 2. The serum levels of interstitial pneumonia markers, such as lactate dehydrogenase (LDH), Krebs von den Lungen-6 (KL-6), and surfactant protein D (SP-D), were not different between the two groups. The forced vital capacity (FVC) was significantly lower in patients with IPPFE than in those with IPF (62.7% vs. 88.6% predicted, p = 0.009), although the diffusion lung capacity for carbon monoxide (DLCO) was not significantly different (p = 0.395). Furthermore, the proportions of neutrophils and eosinophils in the BAL were higher in patients with IPPFE than in those with IPF (2.5% vs. 0.6%, p = 0.018 and 1.7% vs. 0.4%, p = 0.011, respectively).

**Table 2:** Comparison between idiopathic pleuroparenchymal fibroelastosis (IPPFE) and idiopathic pulmonary fibrosis (IPF): laboratory, physiologic, and bronchoalveolar lavage data

	**IPPFE (n = 6)**	**IPF (n = 28)**	**p value**
Laboratory findings			
Serum LDH, U/L	192 ± 37	223 ± 43	0.106
Serum KL-6, U/ml	755 ± 473	1282 ± 888	0.199
Serum SP-D, ng/ml	298 ± 346	222 ± 136	0.527
SP-D/KL-6	0.38 ± 0.20	0.26 ± 0.18	0.168
Physiologic			
Resting PaO_2_, mm Hg	77.6 ± 11.5	83.2 ± 11.0	0.363
Resting PaCO_2_, mm Hg	43.5 ± 7.2	40.6 ± 3.1	0.138
FVC, L	1.97 ± 0.42	2.91 ± 0.91	0.021
FVC, % predicted	62.7 ± 10.9	88.6 ± 21.9	0.009
DLCO, % predicted	77.9 ± 13.4	89.0 ± 26.1	0.395
ΔFVC in 12 mo, L	-0.51 ± 0.35	-0.32 ± 0.38	0.364
BAL fluid cell analysis			
Lymphocytes, %	6.0 ± 4.5	5.2 ± 7.0	0.782
Neutrophils, %	2.5 ± 3.3	0.6 ± 0.8	0.018
Eosinophils, %	1.7 ± 2.0	0.4 ± 0.6	0.011

Data are presented as mean ± SD.

Data are analysed with Student’s t-test.

Definition of *abbreviations*: *FVC* forced vital capacity, *FEV1* forced expiratory volume in 1 second, *DLCO* diffusion lung capacity for carbon monoxide, *BAL* bronchoalveolar lavage.

### Comparison between IPPFE and IPF: HRCT and lung specimen findings

The HRCT and lung specimen findings in patients with IPPFE or IPF are summarized in Table 3. All patients with IPPFE showed upper lobe predominance and pleural thickening on HRCT (p < 0.0001). Higher consolidation scores on HRCT were found in patients with IPPFE than in those with IPF (1.7 vs. 0.3, p = 0.004). No significant differences were found in the other HRCT findings, such as the extent score or ground glass score.

**Table 3:** Comparison between idiopathic pleuroparenchymal fibroelastosis (IPPFE) and idiopathic pulmonary fibrosis (IPF): findings on high resolution computed tomography (HRCT) and lung specimens

	**IPPFE (n = 6)**	**IPF (n = 28)**	**p value**
Findings on HRCT			
Extent score, 0-25	9.8 ± 2.6	8.8 ± 3.7	0.366
Upper lobe predominant distribution, n (%)	6 (100)	0 (0)	<0.0001*
Reticulation score, 0-3	1.3 ± 0.5	1.8 ± 0.7	0.161
Glass score, 0-3	1.5 ± 0.8	1.7 ± 0.7	0.429
Consolidation score, 0-3	1.7 ± 0.8	0.3 ± 0.5	0.004
Existence of honeycombing, n (%)	0 (0)	10 (35.7)	0.148*
Emphysema score, 0-3	0	0.7 ± 0.9	0.078
Pleural thickening, n (%)	6 (100)	2 (7.1)	<0.0001*
Findings on surgical lung biopsy specimens			
Collagen deposition score, 0-3	2.0 ± 0	2.4 ± 0.8	0.175
Existence of microscopic honeycombing, n (%)	1 (16.7)	17 (60.7)	0.078*
Organizing pneumonia score, 0-3	1.8 ± 0.4	0.6 ± 0.7	0.003
Cellularity score in interstitium, 0-3	1.5 ± 0.5	1.3 ± 0.6	0.456
Lymphoid hyperplasia, n (%)	0 (0)	0 (0)	NA.
Pleural fibrosis, pleuritis, n (%)	5 (83.3)	0 (0)	<0.0001*

Data are presented as mean ± SD or, n (%).

Data are analysed with Mann-Whitney’s U-test or *Fisher’s exact probability test.

*HRCT* high resolution computed tomography, *NA* not applicable.

HE-stained lung specimens showed pleural fibrosis or pleuritis underneath the dense subpleural fibroelastosis in five of the six patients with IPPFE (p < 0.0001). Significantly higher organizing pneumonia scores in the alveolar space were found in patients with IPPFE than in those with IPF (1.8 vs. 0.6, p = 0.003). Although the abrupt transition between the subpleural fibroelastosis and underlying normal lung area was more prominent in patients with IPPFE, no significant differences were found in the other pathological findings, such as the collagen deposition score or cellularity score.

### Quantitative analysis of EF in fibrotic lung lesions

**Figure 3:**
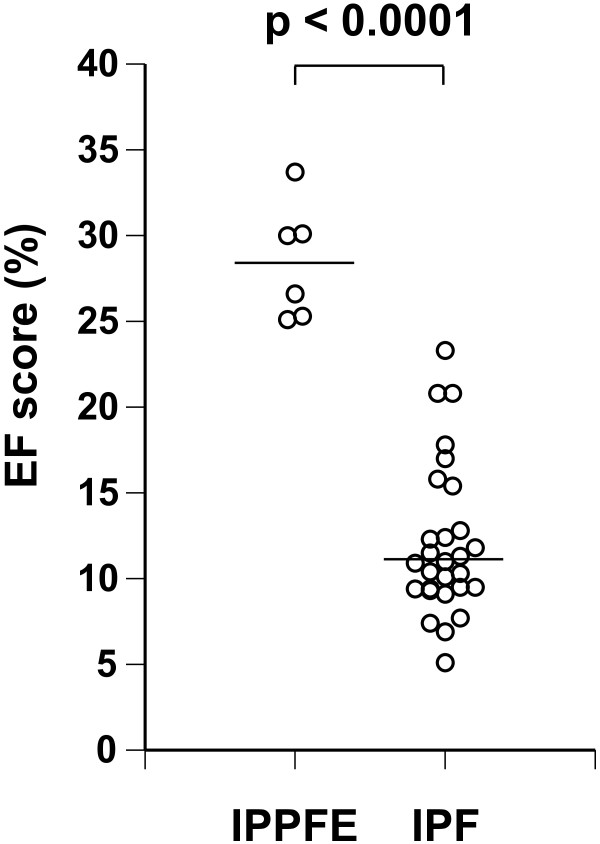
**Total elastic fiber scores of all patients with idiopathic pleuroparenchymal fibroelastosis (IPPFE) or idiopathic pulmonary fibrosis (IPF).** The proportion of elastic fibers (EF score) was calculated by dividing the pixel number of elastic fibers by that of the target fibrotic lung lesion. The horizontal bar represents the median. The median value in patients with IPPFE was 28.3% (range, 25.1-35.6%), and that in patients with IPF was 11.0% (range, 5.1-23.3%). Higher amounts of elastic fiber were seen in the 6 patients with IPPFE than in the 28 patients with IPF (p < 0.0001).

Using a CCD-camera and analytic software, abundant EF were found in the fibrotic lung lesions with EVG staining, and the amount of EF was readily evaluated by our quantitative analytic method (Figure 2). The proportion of EF in fibrotic areas (the EF score) was assessed according to the procedure described in the Methods section. All EF scores are shown in Figure 3. EF scores in patients with IPPFE were significantly higher than those in patients with IPF (median, 28.3% [range, 25.1-35.6%] vs. 11.0% [range, 5.1-23.3%], p < 0.0001). In patients with IPPFE, no correlations were found between EF scores and %FVC (r = -0.058, p = 0.919) (Additional file 1: Figure S1A), between EF scores and %DLCO (r = -0.548, p = 0.384) (Additional file 1: Figure S1B), between EF scores and the change in FVC 12 months after biopsy (r = 0.446, p = 0.631) (Additional file 1: Figure S1C), or between EF scores and the period from detection of interstitial pneumonia until acquisition of lung specimens (r = 0.424, p = 0.433) (Additional file 1: Figure S1D).

### Heterogeneity in the deposition of lung EF in IPPFE

**Figure 4:**
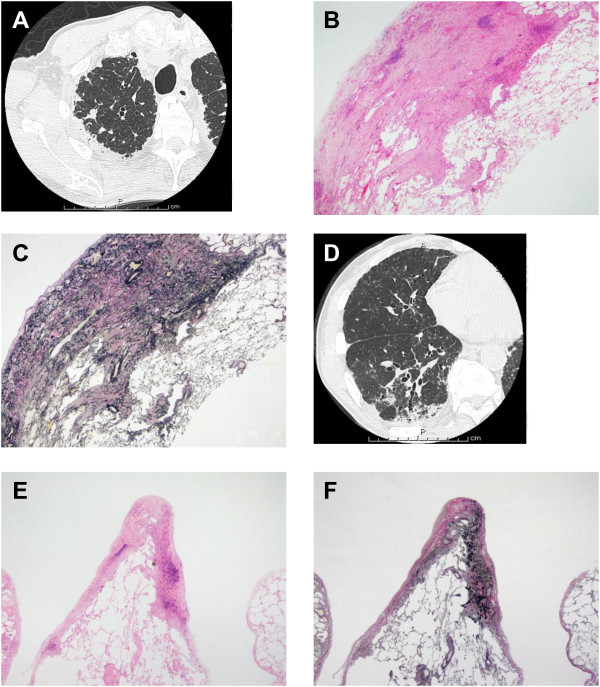
**Comparison of fibroelastosis between the upper and lower lobes in a patient with idiopathic pleuroparenchymal fibroelastosis (IPPFE).** High-resolution computed tomography (HRCT) of the right upper lobe shows pleural thickening and subpleural consolidation opacities **(A)**. A lung section stained with hematoxylin and eosin (HE) shows subpleural fibrosis with an abrupt transition to normal lung parenchyma, similar to usual interstitial pneumonia (UIP) (**B**, ×12.5). A lung specimen stained with Elastica van Gieson (EVG) demonstrates deposition of dense elastic fibers (elastosis) in a subpleural fibrotic lung lesion (**C**, ×12.5). HRCT of the right lower lobe shows pleural thickening and consolidation opacities along a bronchovascular bundle with traction brochioloectasis **(D)**. An HE-stained lung section from the right lower lobe shows a UIP-like lesion (**E**, ×12.5), and a EVG-stained specimen shows fewer elastic fibers in the right lower lobe (**F**, ×12.5) than in the right upper lobe **(C)** in the same patient. In this patient, the elastic fiber score in the lower lobe was 20.3%, whilst that in the upper lobe was 32.4%.

**Figure 5:**
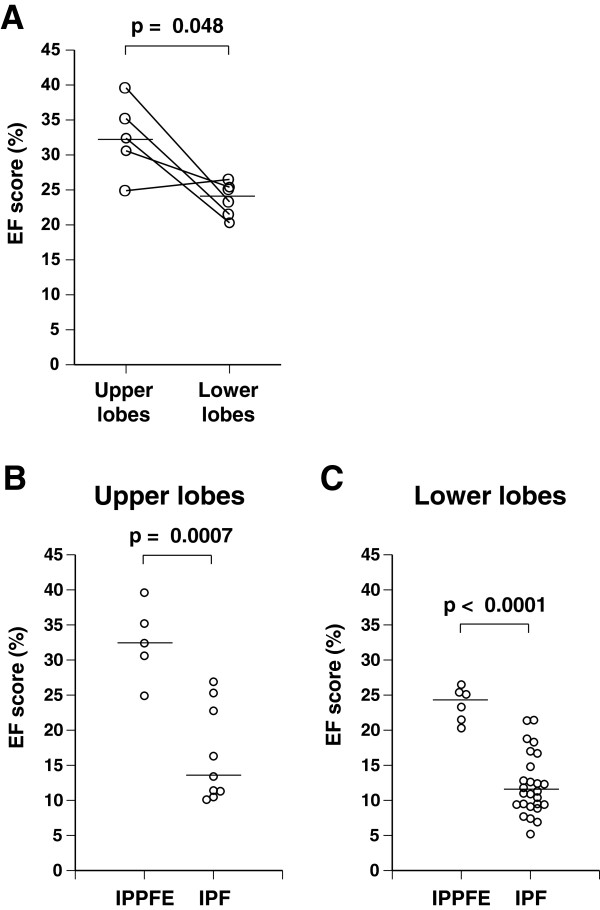
**Heterogeneous deposition of elastic fibers in the lungs of patients with idiopathic pleuroparenchymal fibroelastosis (IPPFE).** Lung specimens were obtained from the both upper lobe and lower lobes in five of the six patients with IPPFE. Elastic fiber scores (EF scores) in each lobe are shown **(A)**. EF scores in lower lobes were significantly lower than those in the upper lobes in patients with IPPFE (median, 24.2% [range 20.3-26.5%] vs. 32.4% [range 24.9-39.6%], p = 0.048). Values in the same patient are connected by solid lines. The horizontal bar represents the median. Even in the lower lobes, the EF scores in patients with IPPFE were still higher than those in patients with IPF (**C**, p < 0.0001) as well as in the upper lobes (**B**, p = 0.0007).

Lung specimens were obtained from two different lobes in five of the six patients with IPPFE. EVG-stained sections showed fewer EF in the right lower lobe (Figure 4D-F) than that in the right upper lobe (Figure 4A-C) in the same patient with IPPFE. In this patient, the EF score in the lower lobe was 20.3% (Figure 4F), whilst that in the upper lobe was 32.4% (Figure 4C). All EF scores in both lobes are shown in Figure 5A. The EF scores in the lower lobes were significantly lower than those in the upper lobes in patients with IPPFE (median, 24.2% [range, 20.3-26.5%] vs. 32.4% [range, 24.9-39.6%], p = 0.048). Conversely, in patients with IPF, the difference in EF scores between the lower lobes and upper lobes was not significant (14.6 vs. 17.2%, p = 0.154) (Figure 5B and C). However, even in the lower lobes, EF scores in patients with IPPFE were still higher than those in patients with IPF (p < 0.0001) (Figure 5C) as well as those in the upper lobes (p = 0.0007) (Figure 5B).

## Discussion

IPPFE was recently classified as a rare IIP in an official ATS/ERS statement [1] and has attracted a great deal of attention. Pathologically, IPPFE shows perilobular collagen deposition with an abrupt transition to underlying normal parenchyma [10]. This finding on HE staining, without elastic staining, is similar to that of IPF/UIP, which is the most common type of IIP and has a grave prognosis [5]. Although some patients with IPPFE have reportedly lived for 7 years or more [2,6], many die before this period [6,11].

As described above, IPPFE has pathological and clinical features similar to those of IPF; however, the precise differences between IPPFE and IPF have not yet been fully elucidated. In this study, a lower incidence of the fine crackles at the time of diagnosis, more pneumothoraces, a higher proportion of never-smokers, and lower body mass indices were seen among patients with IPPFE than with IPF. The lower body mass indices may also have contributed to the lower FVC in patients with IPPFE. Furthermore, the period from detection of interstitial pneumonia to the acquisition of lung specimens was significantly longer in patients with IPPFE than in those with IPF. Although the prognosis of symptomatic patients with IPUF is reportedly poor [11], the prognosis of asymptomatic patients may be relatively better. Otherwise, symptoms may not readily appear in the earlier stage of IPPFE because the FVC before the acquisition of lung specimens was significantly lower in patients with IPPFE than in those with IPF. HRCT and pathological examination revealed more consolidation and more organizing pneumonia in patients with IPPFE than in those with IPF. A diagnosis of IPPFE should be comprehensively worked out using these findings.

Accumulation of dense EF in the subpleural parenchyma is a specific pathological feature of IPPFE [2]. A recent imaging study found a significant increase in the proportion of EF in the alveolar septum in a variety of types of interstitial pneumonia, regardless of their histologic appearance [12]. Basically, collagen fibers and EF accumulate simultaneously in the fibrotic lesions of interstitial pneumonias in humans [12,13], and synthesis and deposition of elastin have been noted in bleomycin-induced interstitial pneumonia in animals [14]. Furthermore, we recently reported that the amount of EF was a significant prognostic factor in patients with IPF [15]. In the present study, more than twice the amount of EF was found in the lungs of patients with IPPFE than in those with IPF by measurement with a CCD camera and analytic software. To our knowledge, this is the first study to quantitatively evaluate the amount of EF in patients with IPPFE. Although the presence of EF is necessary to provide physiological elastic recoil of the lungs, abnormal deposition can adversely alter respiratory movements [16]. Excess amounts of EF affect the “hardness” or “stiffness” of lungs and increase the work of breathing in the early inspiratory phase because of enhanced elastic recoil. There is a possibility that this increase in respiratory workload owing to an excess amount of EF may be related to the lower body mass index and frequent pneumothoraces seen in patients with IPPFE.

Although the prognosis of IPPFE is variable [2], some studies have reported a poor prognosis in patients with IPPFE/IPUF [6,11]. In the present study, three of the six patients with IPPFE died of disease progression, and the decrease in ΔFVC during the 12-month period after biopsy was relatively substantial (-0.51 ± 0.35 L). Although the survival of patients with IPPFE was not significantly different from that of patients with IPF because of the short observation period, it seems that the prognosis of IPPFE is even poorer than that of IPF. A relationship between the amount of EF and the prognosis was not evident in this study. Therefore, parameters with which to predict the prognosis in patients with IPPFE are necessary, and larger studies are required to identify such parameters.

In the present study, significantly lower amounts of EF were found in the lower lobes than in the upper lobes of patients with IPPFE. Reddy et al. also presented a case involving a patient with a lower amount of EF in the lower lobe, which showed a typical UIP-pattern [6]. These findings suggest that typical dense elastosis may be present only in the upper lobes in patients with IPPFE, and biopsy specimens from the lower lobes may contribute to misdiagnosis of IPF/UIP [7]. However, our quantitative study revealed that the amount of EF in patients with IPPFE was still higher, even in the lower lobes, than in patients with IPF. These findings may lead to an accurate histological diagnosis of IPPFE.

The pathophysiology and clinical cause of IPPFE remain unclear. Some patients possess a familial history [2,6], medical history of chemotherapy [2] or recurrent infection [6], lung transplantation [17], or are positive for autoantibodies [6]. Furthermore, overexpression of transforming growth factor-α (TGF-α) may induce progressive interstitial and pleural fibrosis with body weight loss [18,19], and these changes are independent of TGF-β [19]. However, the etiology of IPF is reportedly related to abnormal wound healing in response to multiple microscopic sites of ongoing alveolar epithelial injury [20] and is largely dependent on TGF-β [21]. Therapeutic strategies should differ between the two diseases because of their different pathophysiologies. Therefore, an accurate diagnosis is extremely important to establish novel treatments of each disease in the near future.

Our study has a number of limitations. The number of patients with biopsy-proven IPPFE was extremely small. We hesitated to conduct surgical lung biopsy because of the frequent occurrence of refractory pneumothorax after biopsy [7]. Another limitation is that the study period was relatively short. Finally, this study was conducted retrospectively.

In this study, six patients with IPPFE included only one female. The sex distribution in patients with IPPFE/IPUP is very different between Japan and America/Europe. Many Japanese case reports have described elderly male patients with IPPFE/IPUF [22-24], whilst more patients with IPPFE in America and Europe are younger female [2,6,7]. This may be because of ethnic differences. Additionally, all of these previous papers were case reports or retrospective studies. Therefore, a large prospective and longitudinal cohort study would be the ideal design by which to clarify these issues.

## Conclusions

We found clinical, radiological, and pathological differences between IPPFE and IPF that will help clinicians to properly diagnose IPPFE. Patients with IPPFE exhibited a longer period from detection of interstitial pneumonia to acquisition of lung specimens, a lower incidence of fine crackles, more consolidation on HRCT, and more organizing pneumonia on lung specimens than did patients with IPF. The FVC in patients with IPPFE before biopsy was lower than that in patients with IPF, whilst the decline in FVC after biopsy was comparable with that in patients with IPF. In addition, more than twice the amount of EF was found in the lungs of patients with IPPFE than in the lungs of patients with IPF. Although a lower amount of EF was found in the lower lobes than in the upper lobes among patients with IPPFE, the amount of EF was still higher than that in patients with IPF, even in the lower lobes.

It seems that refractory pneumothorax after surgical lung biopsy of the upper lobes readily occurs in patients with IPPFE [7]. Therefore, we believe that diagnosis of IPPFE should be performed without surgical lung biopsy in the future. The present clinical and radiological findings in addition to some surrogate biological marker data may lead to an accurate diagnosis of IPPFE. Larger studies will be necessary to clarify this.

## Competing interests

The authors declare that they have no competing interests.

## Authors’ contributions

NE, YN, and TS contributed to the study conception and design. HK, YO, MK, YK, SK, DH, TF, KY, NI, YN, and TS analyzed and interpreted the data. NE, YN, and TS drafted the manuscript. All authors read and approved the final manuscript.

## Pre-publication history

The pre-publication history for this paper can be accessed here:

http://www.biomedcentral.com/1471-2466/14/91/prepub

## Supplementary Material

Additional file 1: Figure S1 Relationship between elastic fiber scores (EF scores) and clinical variables in patients with idiopathic pleuroparenchymal fibroelastosis (IPPFE). No correlations were found between EF scores and %FVC (A: r = -0.058, p = 0.919), between EF scores and %DLCO (B: r = -0.548, p = 0.384), between EF scores and the change in FVC 12 months after biopsy (C: r = 0.446, p = 0.631) or between EF scores and the period from detection of interstitial pneumonia to acquisition of lung specimens (D: r = -0.424, p = 0.433). Click here for file
