# Supplementary material for: Quantitative analysis of lung elastic fibers in idiopathic pleuroparenchymal fibroelastosis (IPPFE): comparison of clinical, radiological, and pathological findings with those of idiopathic pulmonary fibrosis (IPF)
Source: BMC Pulm Med. 2014 May 28;14:91. doi: 10.1186/1471-2466-14-91 (PMC4040136; doi:10.1186/1471-2466-14-91)
Supplement: Additional file 1: Figure S1 — Relationship between elastic fiber scores (EF scores) and clinical variables in patients with idiopathic pleuroparenchymal fibroelastosis (IPPFE). No correlations were found between EF scores and %FVC (A: r = -0.058, p = 0.919), between EF scores and %DLCO (B: r = -0.548, p = 0.384), between EF scores and the change in FVC 12 months after biopsy (C: r = 0.446, p = 0.631) or between EF scores and the period from detection of interstitial pneumonia to acquisition of lung specimens (D: r = -0.424, p = 0.433). [file 1471-2466-14-91-S1.pptx]

## Slide 1
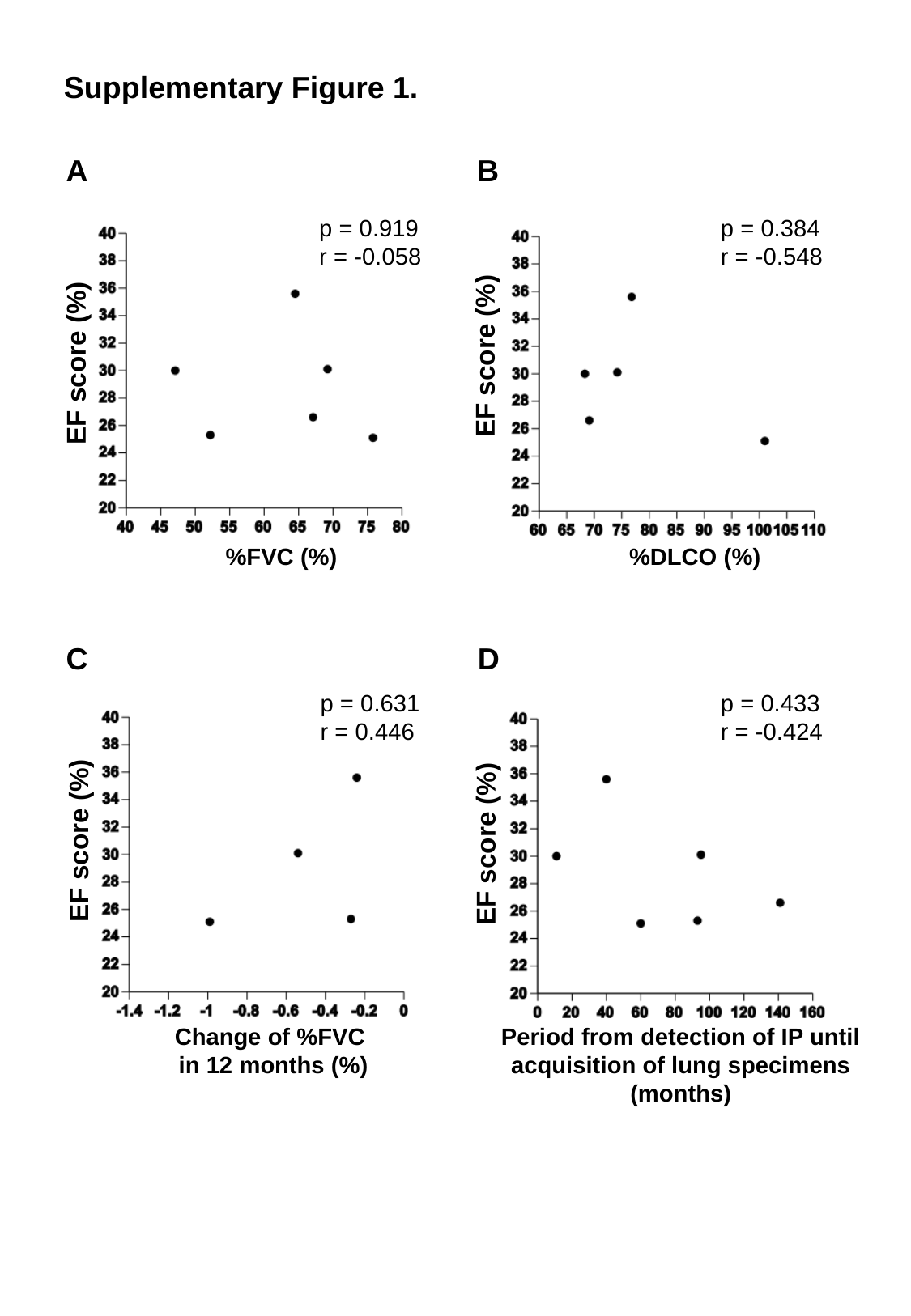

Supplementary Figure 1.
A
B
p = 0.919
r = -0.058
p = 0.384
r = -0.548
EF score (%)
EF score (%)
%FVC (%)
%DLCO (%)
C
D
p = 0.631
r = 0.446
p = 0.433
r = -0.424
EF score (%)
EF score (%)
Change of %FVC
in 12 months (%)
Period from detection of IP until acquisition of lung specimens
(months)
